# Blood parasite infections in a wild population of ravens (*Corvus corax*) in Bulgaria

**DOI:** 10.1186/s12936-018-2179-7

**Published:** 2018-01-16

**Authors:** Peter Shurulinkov, Lachezar Spasov, Georgi Stoyanov, Nayden Chakarov

**Affiliations:** 1 National Museum of Natural History, Bulgarian Academy of Sciences, Sofia, Bulgaria; 2 Birds of Prey Protection Society, Sofia, Bulgaria; 3 Department of Animal Behaviour, Bielefeld University, 33501 Bielefeld, Germany; 4 Molecular Ecology and Evolution Lab, Department of Biology, Lund University, Lund, Sweden

**Keywords:** Avian malaria, *Leucocytozoon*, Host specificity, Corvids, Passerines, Prevalence, Tolerance, Resistance

## Abstract

**Background:**

Blood parasites have been studied intensely in many families of avian hosts, but corvids, a particularly cosmopolitan family, remain underexplored. Haemosporidian parasites of the common raven (*Corvus corax*) have not been studied, although it is the largest, most adaptable, and widespread corvid. Genetic sequence data from parasites of ravens can enhance the understanding of speciation patterns and specificity of haemosporidian parasites in corvids, and shed light how these hosts cope with parasite pressure.

**Methods:**

A baited cage trap was used to catch 86 ravens and a nested PCR protocol was used to amplify a 479 bp fragment of the haemosporidian cytochrome b gene from the samples. The obtained sequences were compared with the MalAvi database of all published haemosporidian lineages and a phylogenetic tree including all detected raven parasites was constructed. An examination of blood smears was performed for assessment of infection intensity.

**Results:**

Twenty blood parasite lineages were recovered from ravens caught in a wild population in Bulgaria. The prevalence of generalist *Plasmodium* lineages was 49%, and the prevalence of *Leucocytozoon* lineages was 31%. Out of 13 detected *Leucocytozoon* lineages six were known from different corvids, while seven others seem to be specific to ravens. A phylogenetic reconstruction suggests that *Leucocytozoon* lineages of ravens and other corvids are not monophyletic, with some groups appearing closely related to parasites of other host families.

**Conclusions:**

Several different, morphologically cryptic groups of *Leucocytozoon* parasites appear to infect corvids. Ravens harbour both generalist corvid *Leucocytozoon* as well as apparently species-specific lineages. The extraordinary breeding ecology and scavenging lifestyle possibly allow ravens to evade vectors and have relatively low blood parasite prevalence compared to other corvids.

## Background

Haemosporidian parasites of the genera *Plasmodium*, *Haemoproteus* and *Leucocytozoon* are widely distributed among birds worldwide and can cause substantial health and fitness reduction to infected hosts [1–4]. Blood parasite prevalence varies greatly among the different families of songbirds *Passeriformes*, and among different species of the same family [5, 6]. Inside the family of warblers (Sylviidae), the highly-parasitized members of the genus *Acrocephalus* share similar habitats with their relatives of the genera *Locustella* and *Cettia* in which haemosporidian infections are of very low prevalence or even absent in some populations [7–9]. Such differences among the bird taxa are difficult to explain. In some cases, they can correlate with different capacities of the host immunity to defend itself against specific parasite infections [10, 11] or with the ecology, habitat preferences and behaviour of the hosts [12–14]. According to studies on different corvid species, such as crows, magpies and jays, the avian family of Corvidae seems to harbour a great amount of haemosporidian parasites [15–18]. However, ravens may be less infected than their relatives due to their specific ecological niche.

The common raven (*Corvus corax*) is the largest songbird and most widely distributed corvid with a range covering nearly the entire Holarctic [19, 20]. Ravens are typical scavengers, often feeding on carrion, with breeding biology resembling vultures and other raptors [21–23]. They breed very early in spring, nesting mostly on rocks in river canyons and mountains, or on trees in lowland and low mountain forests [19]. Particularly in the latter environment they are well exposed to blood parasite vectors—various groups of dipterans. It is hypothesized that scavenging birds are subject to strong parasite-mediated selection on immune defenses, which is supported by the finding that they have larger spleens and higher blood total leukocyte concentrations than non-scavengers [24].

A bird host can utilize two main strategies to cope with an infection—parasite suppression and clearance (resistance), or withstanding the infection, while paying a low fitness cost (tolerance) [25, 26]. In scavenger hosts with particularly potent immunity, parasites may not be able to develop at all. In such species, a very low prevalence of parasites or even their complete absence can be expected. Accordingly, some scavenging birds, such as griffon vultures (*Gyps fulvus*) have very low prevalence of blood parasites [27; own unpublished data]. Other species, such as corvids, harbour a high number of parasites without developing visible illness, corresponding more to a tolerance strategy. Several recent studies have detected high prevalence and high diversity of *Leucocytozoon* parasites in corvids [17, 18, 28]. These studies were completed on close relatives of ravens—crows, jays and magpies, which despite the common incorporation of carrion in their diet are less specialized scavengers compared to ravens. Nevertheless, the phylogenetic relationship between these species allows us to expect similarly high *Leucocytozoon* prevalence in raven populations. Thus, ravens may be expected to have high infection prevalence of blood parasites and low infection intensity.

Until now no haemosporidian genetic lineages from ravens have been studied or are included in MalAvi, a database that includes all published lineages of avian malaria and related blood parasites [29]. The present work, aims to study the prevalence of haemosporidian parasites and their lineage diversity in ravens from a wild population and infer the strategy of ravens to avoid detrimental fitness effects of these parasites.

## Methods

A total of 86 fully grown, adult and immature ravens were sampled in Bulgaria (Dolno Ozirovo, Vratsa district, 43.2393 N, 23.3513 E) between March and September of the years 2013–2016. Ravens were captured in big cage nets baited with carrion, measured and ringed with an ornithological steel ring and a colour ring in order to prevent double sampling. Blood samples were taken from the brachial vein and stored in absolute ethanol. Two blood smears were prepared for each bird and fixed in methanol for 5 min. Smears were stained with Giemsa, 10,000 blood cells scanned and infection intensity score noted according to Valkiūnas [1].

DNA was extracted following a standard phenol–chloroform protocol. For the detection of the blood parasites nested PCR and light microscopy were used. Molecular identification of infections was performed with nested PCR with the primer pairs NaemNFI/NR3, followed by a second reaction with the primers HaemF/R2 and HaemFL/R2L, following the protocol of Hellgren et al. [30]. The PCR amplicons were visualized on a 2% agarose gel with ethidium bromide under UV light. All amplicons from positive samples were purified with ExoSAP and bidirectionally sequenced on an ABI 3730 Analyzer with BigDye Terminator. The cytochrome b sequences were edited and aligned with Geneious 8.1.9 and blasted against the MalAvi database, state 16.07.2017 [29].

Phylogenetic analyses was performed on a dataset of 40 lineages, 20 detected in ravens and close BLAST hits downloaded from the MalAvi dataset. The alignment was trimmed to 464 bp. Trees were constructed using a MrBayes v3.2.6 [31] with a GTR+G model and two Markov chains, run simultaneously for 10 million generations with a subsampling every 200 generations resulting in 50,000 trees. Posterior probabilities were calculated after discarding 25% of burn-in trees. Bootstrap support for individual branches was calculated using 1000 replicates in RAxML [32]. Any sequence differing by one or more nucleotides from other sequences was considered to be distinct. Distinct lineages should not be automatically interpreted as species [18]. Names of the already described parasite lineages are taken from the MalAvi database, while new lineages were designated names CCORAX1–7 (GenBank accession nos. MG209762–MG209768).

## Results

The total prevalence of haemosporidian infections in fully grown ravens determined by molecular methods was 62.8% (Table 1). The prevalence of *Plasmodium*, *Leucocytozoon* and *Haemoproteus* was 48.8% (42/86), 31.4% (27/86) and 2.3% (2/86), respectively. Mixed infections were detected in 16 occasions (29.6% of all infections found; 18.6% of all sampled birds), 15 of them were *Plasmodium*/*Leucocytozoon* and one was *Haemoproteus*/*Leucocytozoon*. A total of 20 haematozoan lineages were detected—*Plasmodium*—5, *Leucocytozoon*—13, and *Haemoproteus*—2. Seven of the *Leucocytozoon* lineages have not been reported until now and have close similarity to other corvid lineages. These lineages have been designated CCORAX1–7 (Table 1).

**Table 1:** Summary of haemosporidian parasite lineages detected in ravens in Bulgaria (n = 86)

GenBank no.	Lineage name	Nr infected	% prevalence	Closest BLAST hit	% match
*Plasmodium* lineages					
AF495571	SGS1	30	34.8		
DQ368381	GRW06	4	4.7		
AY831748	GRW11	3	3.5		
AY831749	SYAT24	3	3.5		
DQ847258	PBPIP	1	1.2		
Total *Plasmodium*		41	47.7		
*Haemoproteus* lineages					
CIRCUM05		1	1.2		
GAGLA05		1	1.2		
Total *Haemoproteus*		2	2.3		
*Leucocytozoon* lineages					
MG209762	CCORAX1	8	9.3	COCOR16	99.6
KJ128991	COCOR13	7	8.1		
MG209765	CCORAX4	2	2.3	COCOR16	99.4
JX867111	COCOR02	1	1.2		
JX867112	COCOR03	1	1.2		
KY768841	PICPIC01	1	1.2		
KJ488810	GAGLA06	1	1.2		
JX507218	EUSE1	1	1.2		
MG209763	CCORAX2	1	1.2	GAGLA06	99.8
MG209764	CCORAX3	1	1.2	COCOR02	97.9
MG209766	CCORAX5	1	1.2	COCOR09	99.8
MG209767	CCORAX6	1	1.2	COCOR16	99.0
MG209768	CCORAX7	1	1.2	CORMAC06	99.8
Total *Leucocytozoon*		27	31.4		
Total infected		54	62.8		
Not infected		32	37.2		

For lineages described for the first time in this study closest BLAST hit to any lineage from the MalAvi database and % similarity are indicated

**Fig. 1:**
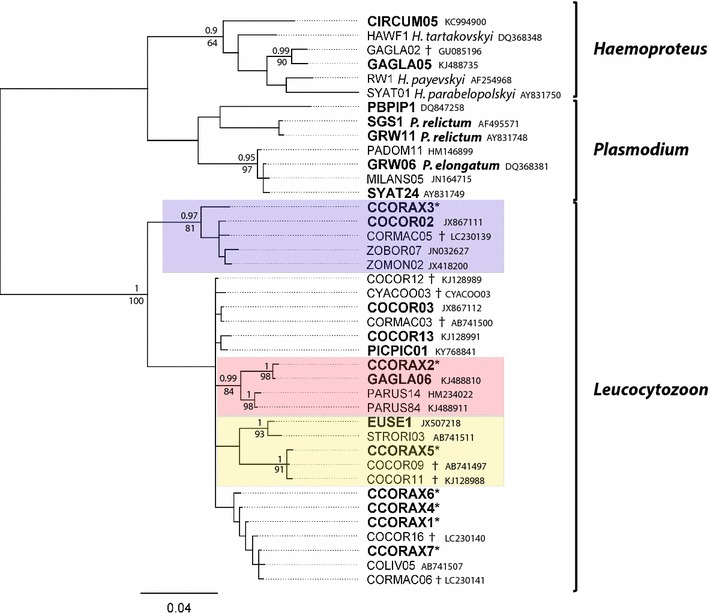
Phylogenetic relationships of lineages detected in ravens (*Corvus corax*) as predicted by Bayesian inference, using GTR+G substitution model in MrBayes v3.2.6 based on 464 bp of cytochrome b sequence. Posterior probabilities are indicated above branch, bootstrap support calculated with RAxML > 70 are below branch. Lineages in bold were recorded in ravens in this study. Lineages reported and named for the first time here are marked with asterisk. Probable clades with high bootstrap support are highlighted. ^†^Indicates corvid parasite lineages closely related to the lineages found in ravens. Graph and support values were produced with Geneious 8.1.9 and edited with Adobe Illustrator

Phylogenetic relationships (Fig. 1) show two main clades inside *Leucocytozoon*—one of them including COCOR02 and CCORAX3, as well as some non-corvid lineages. A second clade contains CCORAX2 and GAGLA06, as well as two *Leucocytozoon* lineages known from blue tits. A third and fourth tentative clades, include CCORAX5, COCOR09, COCOR11, and EUSE1 as well as the lineage STRORI03, known from Oriental turtle doves, respectively. These clades may represent different species of *Leucocytozoon* infecting mostly corvids but also other passerines and doves. A branch including CCORAX1, CCORAX4, CCORAX6 and CCORAX7, recorded in the ravens, as well as COLIV05, CORMAC03, COCOR16 recorded by other authors had low support (0.81 posterior probability and 55% bootstrap support) and could not be verified as a separate clade on the bases of the sequenced cytochrome b region. Several lineages represented in ravens and other corvids such as COCOR12, COCOR13, CORMAC03, CYACOO03 and PICPIC01 do not clearly cluster within any of the distinct clades.

All microscopically detected infections showed very low intensity, below 0.5 parasites/10,000 erythrocytes. *Leucocytozoon* infections were more common during the summer (June–September, 44.9%, 22/49) compared to the spring period (March–May, 18.5%, 5/27, χ^2^ = 5.289, p = 0.021). *Plasmodium* prevalence did not show such seasonal variation.

## Discussion

Ravens in Bulgaria showed relatively low prevalence of blood parasites compared to other studied corvid populations [17, 18, 33, 34]. Although all corvids are scavengers to a different degree, ravens have a very strong expression of this trait. This suggests that ravens may have a particularly potent immunity. Scavengers may have a stronger humoral immune response, potentially effective against malaria-like parasites, but not a stronger cell-mediated immune response [24, 35]. A scavenging lifestyle has also enabled ravens to breed much earlier in spring than crows and magpies, which may allow them to evade the peak of vector emergence [19]. Additionally, raven nests on cliffs are very common at the sampling site and may be less accessible to vectors compared to the nests of other corvids.

Compared to other passerine families, ravens and other corvids have high blood parasite prevalence and are hosts of a number of specific *Leucocytozoon* lineages. This is in accordance with the data presented for three species of North American corvids (*Corvus brachyrhynchos*, *Pica nuttalli* and *Cyanocitta stelleri*) in which high infection prevalence of *Leucocytozoon* infections was reported, and 19 different lineages were found [18].

Almost all microscopically observed infections were of very low intensity, possibly chronic or even latent infections. This suggests that even immature ravens may be past the acute stage of high infection intensity, which most probably occurs in nestlings before fledging. This is the common case in raptors with similar breeding biology, where parental feeding may partly alleviate the risk of starvation during peak infection [1, 36, 37]. The low parasitaemia also suggests that the development of a strong immune response suppresses the blood parasites as ravens get older. Low intensity of infection has been shown to indicate good immune control in several bird species, such as Hawaian amakihi and Seychelles warblers [38, 39]. During the breeding season the parasite suppression may weaken, enabling a relapse, and transmission to new raven hosts [1]. Therefore, both resistance and tolerance are probably dynamic in time.

Among the *Plasmodium* lineages found in the studied ravens, SGS1 (lineage of *Plasmodium relictum*) and GRW6 (lineage of *Plasmodium elongatum*) are wide generalists found in large numbers of hosts [29, Malavi Database 2017]. GRW11 (lineage of *P. relictum*) is also a generalist and can infect a rather wide spectrum of hosts—mostly passerines from different families [6]. It has been found to infect corvids in Asia—Eurasian jays (*Garrulus glandarius*) in Armenia [40], jungle crows (*Corvus macrorhynchos*) in Japan [41] and hooded crows (*Corvus cornix*) in Israel [42]. In Bulgaria GRW11 has been detected in passerines of four families—Ploceidae, Fringillidae, Sylviidae and Laniidae [43–45]. The lineage SYAT24 until now was found only in blackcaps (*Sylvia atricapilla*) migrating through Spain [46, 47]. Blackcap is a common breeding and migrating bird in the area of the raven’s samples origin for the present study (Dolno Ozirovo). Probably SYAT24 has been transmitted from blackcaps to the local ravens in Bulgaria. Similarly, the lineage PBPIP1 occurs mostly in African birds (local residents) and European long distance migrants—tree pipits (*Anthus trivialis*) and collared flycatchers (*Ficedula albicollis*), wintering in Africa (Malavi Database 2017). Since these migratory species regularly pass through the study area, most likely the lineage has been transmitted locally in Bulgaria to the ravens. Thus, some local mosquitoes are probable competent vectors for this *Plasmodium* lineage.

*Plasmodium* prevalence in corvids appears to follow a latitudinal gradient. This can be expected for parasites of non-migratory species, which depend on suitable climatic conditions for their vectors [1, 48]. *Plasmodium* was not found in crows of two species (*Corvus corone*, *C. macrorhynchos*) in Hokkaido, Japan [28]. Most probably this northern island lacks suitable conditions for competent vectors. However, another study from southern Japan showed 17% prevalence of *Plasmodium*/*Haemoproteus* in the same two species [33]. A population of carrion crows in Germany had a *Plasmodium* prevalence of 29.5% [17]. A high prevalence of 59.6% *Plasmodium*/*Haemoproteus* infections has been reported for hooded crows (*C. cornix*) in Italy [34], which is even higher than the 48.8% *Plasmodium* prevalence for ravens in Bulgaria.

The *Haemoproteus* lineages GAGLA05 and CIRCUM05, which were detected during the present study in one raven each, have been reported from jays (*G. glandarius*) sampled in Portugal and Morocco [6]. In this case, despite not appearing closely related, both haemoproteid lineages infect birds belonging to two different genera of corvids, a pattern strongly expressed by *Leucocytozoon*.

The prevalence of *Leucocytozoon* found in ravens from Bulgaria was much lower compared to the other studied corvid species (Table 2). The phylogeny of raven-infecting *Leucocytozoon* suggests at least two distinct clades. The parasite lineages CCORAX3 and COCOR02 form a clade with the non-corvid lineages ZOMON02 and ZOBOR07 of white-eyes (*Zosterops*). The majority of corvid *Leucocytozoon* lineages do not cluster significantly in a clade. The lineage CCORAX2 is closely related to GAGLA06 which was reported from Eurasian jays (*G. glandarius*) sampled in Portugal [6]. Both lineages seem to be closely related to the lineages PARUS14 and PARUS84 infecting blue tits (*Cyanistes caeruleus*). Obviously, ravens share some haemosporidian lineages with other European *Corvus* species but also with species of other corvid genera. However, a phylogeny based on this cytochrome b fragment does not suggest a single clade of purely corvid-infecting *Leucocytozoon*. Although it was previously known only from blackfly vectors, the results of this study show that the lineage EUSE1 can infect ravens [49]. This lineage forms a clade with the *Leucocytozoon* lineage STRORI03, known from doves, rather than with other corvid lineages. The lineages COCOR3 and COCOR13 were found in hooded crows (*C. cornix*) and domestic pigeons (*Columba livia f. domestica*) in Italy [16, 34]. In this case, the same leucocytozoid lineage was found in birds of different orders living in similar habitat. Both latter cases suggest that corvids and doves may have similar host characteristics, predisposing for host switching. Indeed, *Leucocytozoon* have recently been shown to have high rates of co-speciation with their hosts but also high rates of host switching [50].

**Table 2:** *Leucocytozoon* parasites prevalence in different studied birds of family Corvidae (only results of molecular studies included)

Corvid species	Location	Prevalence	N	Source
*Corvus corone*	Hokkaido, Japan	93.2	117	Yoshimura et al. [28]
*Corvus cornix*	Italy	97.8	46	Scaglione et al. [34]
*Corvus macrorhynchos*	Hokkaido, Japan	95.8	24	Yoshimura et al. [28]
*Corvus corone*	Germany	85.3	85	Schmid et al. [17]
*Corvus brachyrhynchos*	California, USA	57.4	258	Freund et al. [18]
*Pica nuttalli*	California, USA	54.5	44	Freund et al. [18]
*Cyanocitta stelleri*	California, USA	100.0	20	Freund et al. [18]
*Corvus corax*	Dolno Ozirovo, Bulgaria	31.4	88	Present study

The present study shows that even in widely distributed scavenger hosts with relatively low prevalence, such as ravens, the variety of *Leucocytozoon* lineages is quite high. The results also show that although most *Leucocytozoon* lineages in corvids are closely related, as pointed out by Freund et al. [18], they are represented by at least two distinct clades, both of which contain lineages probably specialized on corvids and other host groups, along with lineages tending toward a broader host spectrum.

## Conclusion

Several distinct clades of *Leucocytozoon* parasites appear to infect corvids. Ravens harbour both generalist corvid *Leucocytozoon* as well as apparently species-specific lineages. The extraordinary breeding ecology and scavenging lifestyle possibly allows ravens to evade vectors and have relatively low blood parasite prevalence compared to other corvids.
